# Distinct Role of *Lycium barbarum* L. Polysaccharides in Oxidative Stress-Related Ocular Diseases

**DOI:** 10.3390/ph16020215

**Published:** 2023-01-31

**Authors:** Yali Niu, Guoheng Zhang, Xiaojia Sun, Shikun He, Guorui Dou

**Affiliations:** 1 College of Life Sciences, Northwestern University, Xi’an 710069, China; 2 Department of Ophthalmology, Eye Institute of Chinese PLA, Xijing Hospital, Fourth Military Medical University, Xi’an 710032, China; 3 Department of Ophthalmology, USC Roski Eye Institute, Keck School of Medicine, University of Southern California, Los Angeles, CA 90033, USA

**Keywords:** *Lycium barbarum* polysaccharides, antioxidant, oxidative stress, diabetic retinopathy, hypertensive neuroretinopathy, age-related macular degeneration, retinitis pigmentosa

## Abstract

Oxidative stress is an imbalance between the increased production of reactive species and reduced antioxidant activity, which can cause a variety of disturbances including ocular diseases. *Lycium barbarum* polysaccharides (LBPs) are complex polysaccharides isolated from the fruit of *L. barbarum*, showing distinct roles in antioxidants. Moreover, it is relatively safe and non-toxic. In recent years, the antioxidant activities of LBPs have attracted remarkable attention. In order to illustrate its significance and underlying therapeutic value for vision, we comprehensively review the recent progress on the antioxidant mechanisms of LBP and its potential applications in ocular diseases, including diabetic retinopathy, hypertensive neuroretinopathy, age-related macular degeneration, retinitis pigmentosa, retinal ischemia/reperfusion injury, glaucoma, dry eye syndrome, and diabetic cataract.

## 1. Introduction

Reactive oxygen species (ROS) is a general term for oxygen-containing free and non-free radicals produced during the body’s metabolic process [1]. Although ROS is a byproduct of cell metabolism, cells need a certain amount of ROS to maintain body balance under normal physiological conditions [2]. Many studies have shown that ROS plays a vital role in biological processes such as cell proliferation and differentiation, gene expression, protein modification, cell signaling, and destruction of intracellular pathogens [3,4,5]. Excessive ROS production leads to oxidative stress, an imbalance between the increase in active species and the decrease in antioxidant activity [6]. These excess oxygen radicals can cause damage to macromolecules such as DNA, proteins and lipids, leading to a variety of oxidative stress-related diseases [7,8]. Studies have reported that more than 100 diseases are related to oxidative stress; they include neurodegenerative diseases, cancer, kidney disease, cardiovascular disease, diabetes, ocular degenerative diseases, autoimmune rheumatic diseases, and inflammatory diseases [9]. The eye is the primary light-sensitive organ exposed to various ultraviolet and visible light. Oxidative stress is exacerbated in the eye, damaging ocular tissues and functions, increasing vascular permeability, and eventually leading to microvascular abnormalities and neovascularization as well as various vision-threatening diseases such as age-related macular degeneration (AMD), cataract, dry eye syndrome (DES), retinitis pigmentosa (RP), and diabetic retinopathy (DR) [10,11].

Antioxidants are effective in preventing damage to the body from these free radicals [12]. However, many synthetic antioxidants are shown to be toxic and should not be used in large quantities for a long time [13]. Therefore, screening for natural and harmless antioxidants became an urgent demand. Many natural plant ingredients have been shown to possess antioxidant effects, particularly plant polysaccharides [14]. In recent years, natural plant polysaccharides have undertaken a significant role in the research of antioxidant drugs. They are proved to be reasonably effective in preventing oxidative stress caused by excess free radicals [15,16]. *Lycium barbarum* polysaccharides (LBPs), which are natural polysaccharides extracted from the fruits of *L. barbarum*, have been extensively studied [17]. LBPs nourish the eyes and liver in the traditional concept of Chinese medicine. Several studies explored the various functions of LBPs, such as immunity enhancement, anti-tumor activities, antioxidation, blood sugar and lipid regulation, and protection against radiation, and are promising as natural antioxidants [18]. In particular, the antioxidant effect of LBP has been extensively studied. Significant progress has been made in the study of the antioxidant mechanism of LBP and its application in ophthalmic diseases in recent decades. In this paper, we analyzed and reviewed the studies on the antioxidant effect of LBP in DR, hypertensive neuroretinopathy, AMD, RP, retinal ischemia/reperfusion injury, glaucoma, DES and diabetic cataract, by reviewing the recent studies on LBP at home and abroad, to provide new ideas and methods for the subsequent research on LBP. It also provides the theoretical basis for the early clinical application of LBP.

## 2. The Natural Existence and Acquisition of LBPs in Daily Life

### 2.1. Natural Existence of LBPs

**Figure 1:**
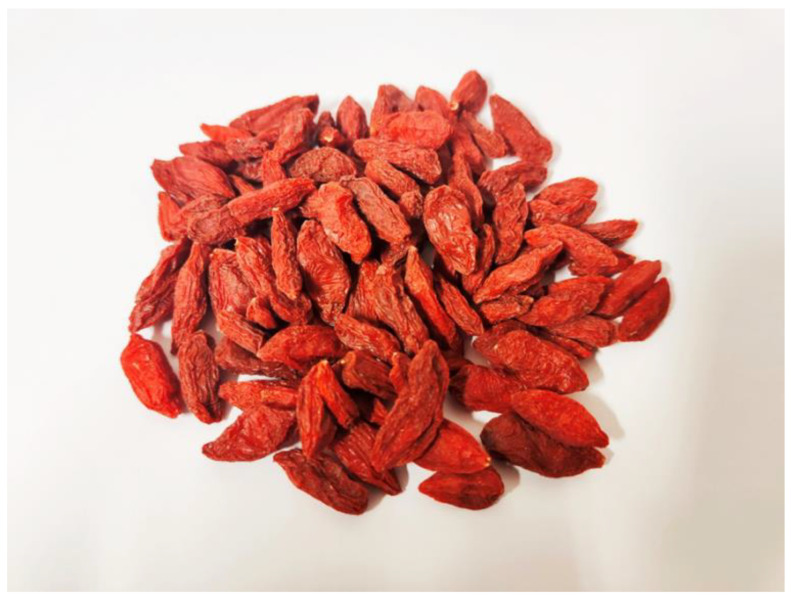
The dried fruit of *Lycium barbarum*.

*L. barbarum* L. (wolfberry or goji berry) (see Figure 1) is a perennial shrub belonging to the Solanaceae family, mainly distributed in East Asia, especially in South China, Korea, and Japan [19]. There are 80 species belonging to the genus *Lycium* globally, while only three of them (*L. barbarum*, *L. Chinese*, and *L. ruthenicum*) are used for medical purposes, known as goji berries in China. *L. barbarum* accounts for almost 90% of available goji berries in the market and has been widely cultivated in northwest China for more than 600 years [20]. As a nutritious and edible medicinal plant, goji berry has been used as a traditional Chinese medicine and food supplement in China for more than 2000 years [21]. *L. barbarum* is rich in polysaccharides, carotenoids, phenols, alkaloids, amines, betaine, essential oils, anthocyanins, trace elements, amino acids, and vitamins, among which LBPs are the main components accounting for 5–8% of the dried fruit [22,23].

### 2.2. Extraction and Biological Structure of LBPs

**Figure 2:**
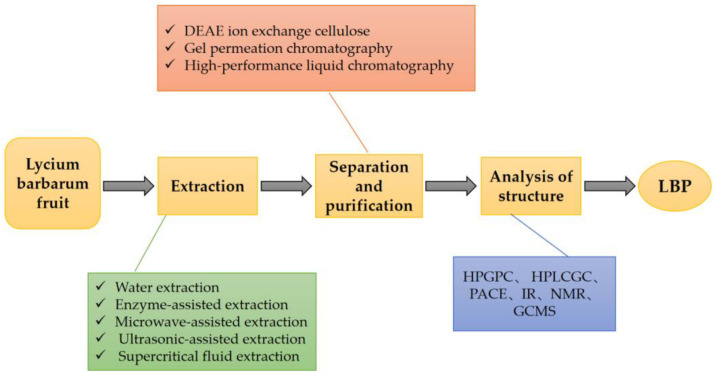
The process of extraction, separation, and purification of LBPs. LBPs can be obtained by extraction, separation, purification, and structural analysis from *L. barbarum* fruit. DEAE: Diethylaminoethyl, HBGPC: high performance gel permeation chromatography, HPLC: high performance liquid chromatography, GC: gas chromatography, PACE: polyacrylamide co-electrophoresis, IR: infrared, NMR: nuclear magnetic resonance, GCMS: gas chromatographic mass spectrometry.

LBPs are derived from the extracts of the fruits of *L. barbarum* [18]. There are various methods for extracting LBPs, including the traditional water extraction method, enzyme-assisted method, microwave-assisted method, ultrasonic-assisted method, and supercritical fluid extraction method [24]. In all these extraction methods, the similar underlying principle is the degradation of the cell wall under certain conditions without affecting the polysaccharide activity [25]. Each extraction method has its pros and cons [26]. When selecting specific methods, it is essential to weigh the high extraction rate and biological activity of LBPs. There have been several reports of quantitative data on the polysaccharide content in Fructus lycii. A study determined the mass fraction of polysaccharides in dried *L. barbarum* as 23% [27]. Another study separated and purified LBPs and analyzed their structure [24]. The detailed process and method are shown in Figure 2**.**

**Figure 3:**
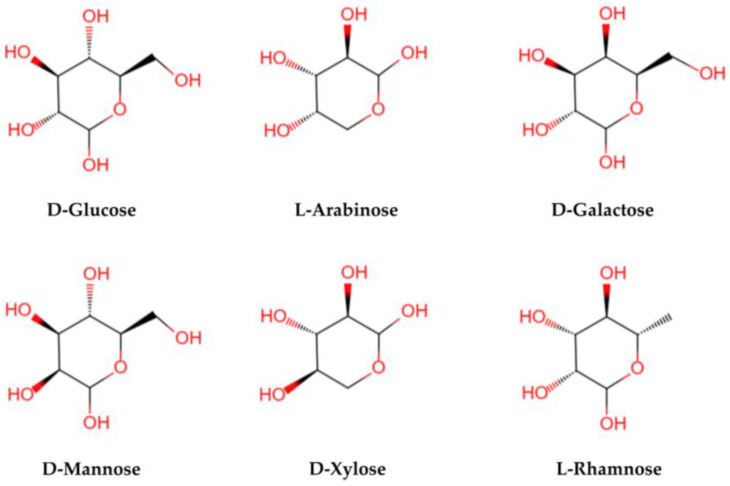
The structural formula of main monosaccharides in LBPs. LBPs mainly contain six monosaccharides (glucose, arabinose, galactose, mannose, xylose, and rhamnose).

LBPs are glycopeptide chains consisting of acidic heteropolysaccharides and polypeptides or proteins. Polysaccharides are characterized by a complex structural hierarchy, with structure levels classified as primary, secondary, tertiary, and quaternary. Their advanced structures are based on their primary structures. The structure and composition of LBPs are different because of the different methods of separation and purification. The relative molecular mass is generally 10–2300 KD [18]. Structurally, LBPs contain six monosaccharides (arabinose, glucose, galactose, mannose, xylose, and rhamnose) (see Figure 3), galacturonic acid, and 18 amino acids. Monosaccharides are linked by glycosidic bonds that are mainly (1→3)-β-Galp, (1→4)-β-Galp, (1→4)-β-Galp, (1→6)-α-glucans, and (1→4)-α polygalacturonans with various branches and terminals [28,29]. The LBPs are highly branched, and the main chain is a (1→6) Galp-connected galactose [30]. It is the complex structure of a polysaccharide that determines its biological activity [26].

Polyphenols are also notable active ingredients in *L. barbarum*. At present, 53 kinds of polyphenols have been identified from *L. barbarum*, including 28 kinds of phenylpropane, 4 kinds of coumarins, 8 kinds of lignans, 5 kinds of flavonoids, 3 kinds of isoflavones, 2 kinds of chlorogenic acid derivatives, and 3 kinds of other components [31]. They showed strong oxygen radical absorption capacity and DPPH radical scavenging activity. There are many extraction methods for polyphenols. More and more green and sustainable extraction techniques have been discovered and applied [32,33]. At present, ultrasonic extraction, microwave extraction, enzyme-assisted extraction, and solvent extraction have been employed to extract polyphenols [34,35,36].

## 3. Biological Function of LBPs

### 3.1. Anti-Oxidative and Anti-Aging Effects

Stimulation by external factors induces excessive free radical production, leading to various diseases such as arthritis, atherosclerosis, cancer, chronic inflammation, diabetes, and senescence [37]. Several studies have validated that LBPs possess antioxidant and anti-aging properties attributed to the presence of carotenoids, ascorbic acid and their derivatives, flavonoids, and other phenolic compounds. The mechanisms include the direct or indirect scavenging of free radicals, lipid peroxidation inhibition, and the maintenance of cell membrane and macromolecular structure [18]. Zhang et al. [38] extracted L. barbarum seed dreg polysaccharides using four different solvents. They observed that concentrated alkaline preferred to scavenge 2,2-diphenyl-1-picrylhydrazyl (DPPH) free radicals, while hot buffer scavenged OH free radicals and the chelating agent (CHSS) chelated ferrous ions. They also found that polysaccharides had good antioxidant activities. Wang et al. [39] reported that LBPs extracted by different methods presented a solid ability to scavenge DPPH, hydroxyl free radicals, and superoxide anion radicals. In addition, LBPs could prolong the life span of nematodes under normal conditions, oxidative stress, and heat stress. The anti-aging role of LBPs by upregulating the expressions of daf-16, sod-3, and hsp-16.2 genes might be responsible for this. In addition, LBPs could reduce the expressions of age-related genes p21 and p53 in zebrafish embryos, thereby regulating the p53 signaling pathway to delay aging [40].

### 3.2. Immune Regulation

The immunomodulatory effect of LBPs has been widely studied. Research indicated that LBPs activated dendritic cells, macrophages, T and B lymphocytes, natural killer cells, and other immune cells to regulate the immune response in the body [41]. Zhang et al. [42] found that the *Lycium barbarum* polysaccharide LBPF4-OL might promote lymphocyte proliferation in wild-type (C3H/HeN) mice and induce the production of TNF-α and IL-1β in peritoneal macrophages isolated from the mice; LBPs functioned as immunomodulators. Bo et al. [43] showed that LBPs facilitated the maturation of dendritic cells; upregulated the expressions of MHCII, CD80, and CD86; and promoted antigen uptake. In addition, Su et al. found that LBPs upgraded the activation of follicular helper T cells and induced IL-21 secretion to enhance humoral immunity [44]. Sulfated LBPs can significantly enhance the immune activity of cultured chickens [45].

### 3.3. Neuroprotective Effects

LBPs were reported as neuroprotective. In ischemic nerve injury, LBPs inhibited the NR2B signaling pathway while activating the NR2A signaling pathway to play a neuroprotective role [46]. In a diabetic rat model, LBP treatment significantly increased the number of retinal ganglion cells (RGCs) and amacrine cells. The function of neuroprotection might be related to the activation of the Nrf2/HO-1 antioxidant pathway [47]. Yu et al. [48] showed that LBPs prevent oxygen-glucose deprivation/reperfusion-induced neuronal damage in primary hippocampal neurons by activating the PI3K/Akt/mTOR signaling pathway.

### 3.4. Anti-Tumor Effect

The antitumor effects of LBPs are mainly achieved by inhibiting the growth of tumor cells, promoting the apoptosis of neoplastic cells, enhancing the immune function of host cells, and reducing toxicity in combination with chemotherapy drugs [49]. Gong et al. [50] found that the component of LBPs, LBGP-I-3 (arabinogalactan fraction), possesses the most substantial inhibitive effect on the growth of hepatoma cells (SMMC-7721), cervical cancer cells (HeLa), and human breast cancer cells (MCF-7 cells). LBGP-I-3 facilitated the arrest of the MCF-7 cell cycle during the G0/G1 phase, which reduced the apoptosis-associated protein Bcl2/Bax ratio and increased the expressions of Caspase-3, 8, and 9 and ROS production. Additionally, mitochondrial membrane potential decreased with regulated expressions of phosphorylated Erk, JNK, and p38 proteins. Further, Deng et al. [51] observed that LBP-3 had the highest inhibitory efficiency, which could induce cell apoptosis, mitochondrial membrane potential destruction, and the arrest of mouse liver cancer cells, H22 cells in S-phase in vitro, which was confirmed in H22 tumor-bearing mice [52]. Furthermore, Zhang et al. [53] studied the effects of LBPs on the proliferation and apoptosis of the QGY7703 human hepatocellular carcinoma cells. It was found that the apoptosis of the cells treated by LBPs increased, accompanied by an increase in Ca^2+^ ions. It indicated that LBPs increased the concentration of calcium ions in cells, which was closely related to the signal transduction pathway of apoptosis, and affected the chemical sensitivity of tumor cells to anticancer agents. In addition, the combination of LBP with doxorubicin enhanced the antitumor activity of doxorubicin, increased the peripheral blood lymphocyte count, and promoted the cell cycle recovery of bone marrow cells [54].

### 3.5. Anti-Inflammatory Effect

LBPs have satisfactory anti-inflammatory effects and have been used to treat alcoholic liver disease, hepatitis, diabetes, kidney disease, and other disorders. Liao et al. [55] induced a renal injury model through a high-fat diet and treated the mice with oral administration of LBPs. It showed that the administration of LBPs restored the concentrations of lipid, blood urea nitrogen, serum creatinine, and urine protein. Additionally, the concentrations of SREGBP-1, TNF-α, and IL-6 were downregulated. In contrast, the concentrations of adiponectin and AMPK were upregulated in the kidney, which indicated that LBPs could enhance the anti-inflammatory responses and alleviate renal injury caused by lipid metabolism disorder. Xiao et al. [56] found that LBPs treatment of non-alcoholic steatohepatitis mice reduced liver inflammation and cell apoptosis, which might be achieved through the regulation of autophagy and the MAPK pathway. Du et al. [57] found that LBPs significantly inhibited albuminuria and reduced the blood urea nitrogen concentrations, as well as the expressions of serum inflammatory factors, including IL-2, IL-6, TNF-α, IFN-α, MCP-1, and ICAM-1 in diabetic rats, which indicated the anti-inflammatory properties of LBPs in diabetes mellitus.

### 3.6. Radiation Protection

Prolonged exposure to all types of radiation could cause severe damage to the skin, eyes, and nervous system, as well as accelerated aging. Plant polysaccharides have potential anti-radiation effects through antioxidation and cell protection. On the one hand, LBPs assume antioxidant roles in reducing oxidative stress. On the other, they reduce apoptosis and improve the survival of damaged cells [37]. Liang et al. [58] found that LBPs could improve UV-induced cell damage by boosting nuclear Nrf2 phosphorylation in HSF cells. Li et al. [59] found that LBPs pretreatment significantly attenuated the UVB-induced decrease in cell viability, ROS production, and DNA damage with immortalized human keratinocytes (HaCaT cells). LBPs have also been reported to protect cells from UV damage by increasing the concentrations of superoxide dismutase (SOD) and glutathione peroxidase (GSH-Px) [60].

### 3.7. Hypolipidemic and Hypoglycemic Effects

Several studies have shown that LBPs could reduce blood sugar and dyslipidemia. This may be achieved by improving islet cell function and promoting their regeneration, improving insulin sensitivity, and increasing the expression of antioxidant enzyme genes [61]. Zhu et al. [62] found that LBPs promoted the proliferation of pancreatic cells and the release of insulin by RIN-m5f, a pancreatic cell line, which facilitated hypoglycemia. In a high-fat diet and streptozotocin-induced diabetic rat model [63], treatment with LBPs for 30 days significantly reduced fasting blood glucose and increased the concentration SOD. In addition, Li et al. [64] found that LBPs significantly reduced the blood glucose concentration of mice induced by a high-fat diet and triglyceride (TG) and DAG concentrations in serum and liver by regulating AMPK activity in hepatocytes and inhibiting the expression of SREBP-1c. Yang et al. [26] reported that LBPs reduced the concentration of glucose in the blood and alleviated insulin resistance in obese mice, which, in turn, reduced the serum concentrations of TG, total cholesterol (TC), and low-density lipoprotein cholesterol (LDL-C) and increased that of high-density lipoprotein cholesterol (HDL-C). Meanwhile, the expressions of SOD and GSH increased while that of malonic dialdehyde (MDA) decreased. These results indicate that LBPs reduce blood glucose and lipid concentrations.

### 3.8. Regulation of Intestinal Flora

The human gut is home to more than 500 microorganisms that are involved in the body’s absorption and replacement, as well as the development of various diseases such as bowel cancer, obesity, type 2 diabetes, and autoimmune diseases [65,66]. Several studies showed that LBPs regulated intestinal flora and improved various functions of the body. Zhu et al. [67] found that LBPs treatment increased the growth of the probiotics Lactobacillus acidophilus and bifidobacterium longum in Man Rogosa Sharpe broth in vitro. In vivo, the administration of LBPs in mice increased the abundance of Proteobacteria and Firmicutes at the phylum level and promoted the emergence of probiotics, such as Akkermansia, Lactobacillus, and Prevotellaceae, at the genus level [67]. In short, LBPs affected the intestinal microbial community of mice and promoted the growth of beneficial bacteria. Zhao et al. [68] also proved that LBPs treatment increased the intestinal microbial diversity of rats with chronic prenatal stress and attenuated the emotional sadness of their offspring. In a high-fat diet/streptozotocin-induced diabetic mouse model, oral administration of LBPs for 6 weeks increased the number of microbes in the taxa of the genus Allobaculum involved in glycemic control, indicating that LBPs played an anti-diabetic role by altering the gut microbiome [69]. Cao et al. [70] found that the administration of LBP-3 (Arabingalactan) might alleviate DSS-induced chronic colitis by enriching potential probiotics (Rumenococci) and inhibiting the proliferation of harmful bacteria (Enterobacteriaceae).

### 3.9. Anti-Viral Activity

**Figure 4:**
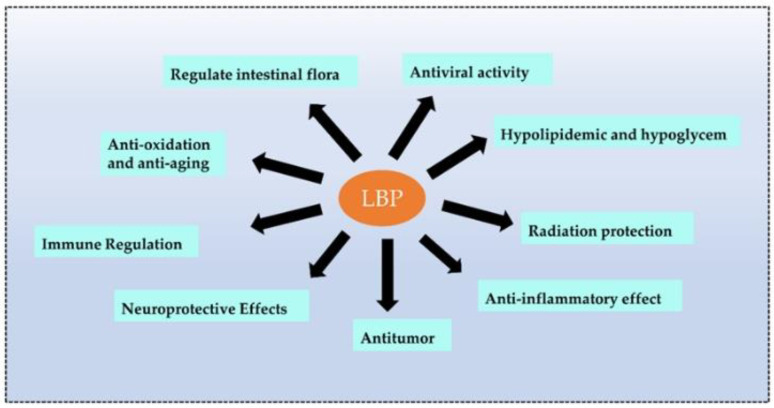
The main biological functions of LBPs. LBPs have many functions, and the main functions are listed above.

Recent studies revealed that plant polysaccharides could inhibit viruses and were more environmentally friendly and safer than antibiotics. Polysaccharides are biological macromolecules that effectively encapsulate viruses, preventing them from entering and attacking cells. LBPs also enhanced immune function and antiviral effects [71]. Wang et al. [72] carried out four types of sulfated modifications on extracted LBPs and found that LBPs prevented infections of the Newcastle virus in chicken embryo fibroblasts (CEF). The sulfated LBPs could exhibit higher inhibitory efficiency. Bo et al. [73] wrapped LBPs in liposomes and observed the promotion of splenic cell proliferation in vitro, a significant increase in the ratio of CD4+ T to CD8+ T cells, and the promotion of the secretion of macrophage cytokines. Moreover, LBPs significantly enhanced the immune response to the PCV2 vaccine in vivo [73] (the biological function of LBPs is shown in Figure 4).

## 4. Molecular Mechanisms Related to the Antioxidant Effect of LBPs

**Figure 5:**
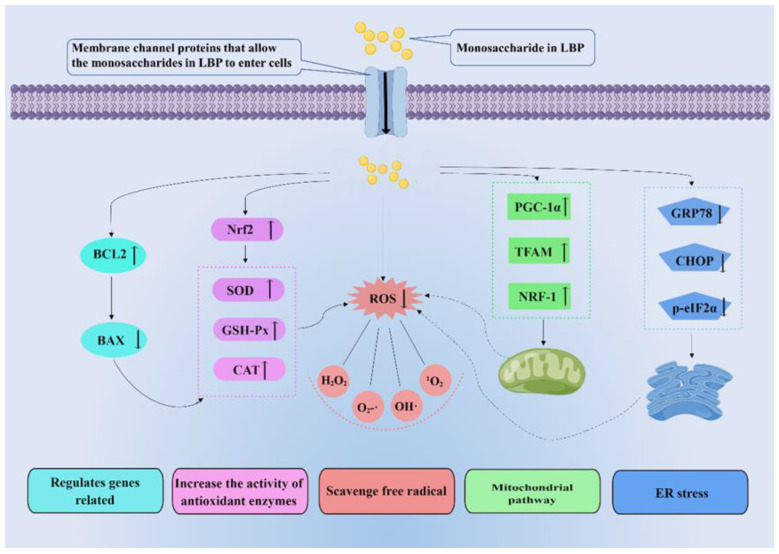
Molecular mechanisms related to the antioxidant effect of LBPs. LBPs play antioxidant roles by directly or indirectly eliminating free radicals. Indirect ways of scavenging free radicals include regulating related genes, increasing the activity of antioxidant enzymes, mitochondrial pathway, and endoplasmic reticulum (ER) stress. Nrf2: nuclear respiratory factor 2; GSH-Px: glutathione peroxidase; CAT: catalase glutathione; ROS: reactive oxide species; PGC-1α: peroxisome proliferators-activated receptor γcoactivator-l alpha; TFAM: transcription factor A, mitochondrial; NRF-1: nuclear respiratory factor 1; GRP78: glucose regulated protein 78; CHOP: C/EBP homology protein; p-eIF2α: phosphorylated eukaryotic initiation factor-2 alpha.

Anti-oxidation mitigates free radicals. The body has a redox system to balance the production of ROS. Antioxidants, substances that inhibit oxidation, are thought to protect against oxidative stress [74]. Antioxidants mainly consist of the following natural enzymes: SOD, catalase (CAT), and GSH-Px [75]. SOD is an antioxidant metal enzyme that can decompose superoxide into oxygen and hydrogen peroxide and reduce the peroxide effect of superoxide on lipids [76]. In addition, SOD is essential for activating CAT and GSH-Px. GSH-Px is a ubiquitous antioxidant enzyme that converts peroxides into non-toxic oxygen and water. It can also individually trap free radicals and convert them to non-toxic forms [77]. CAT further converts H2O2 to water and prevents the formation of hydroxyl radicals [76]. LBPs can improve the activity of these enzymes to achieve antioxidant effects. Therefore, research on antioxidants provides insights of great import into the prevention and treatment of various redox-related diseases [78]. In recent years, the antioxidant effects of plant polysaccharides have been widely studied. LBPs, the natural compounds, have been shown to exhibit antioxidant activity in several studies. LBPs may perform antioxidant roles by eliminating free radicals directly or indirectly (Figure 5).

### 4.1. LBPs Scavenge Free Radicals

There are four types of free radicals, including carbon, nitrogen, oxygen, and sulfur. Oxygen-containing free radicals are the most common [79]. The principal free radicals include hydrogen peroxide (H_2_O_2_), superoxide radical (O2•−), hydroxyl radical (OH•), singlet molecular oxygen (1O_2_), and lipid peroxide [80]. Polysaccharides may be antioxidative in the following ways. (1) Polysaccharides may break down free radicals into non-toxic substances by directly binding to them. (2) Polysaccharides can bind with metal ions, weaken lipid peroxidation, and inhibit free radical formation [81]. Sulfated polysaccharides seem to have stronger effects on free radical scavenging [81]. The direct scavenging of free radicals by LBP to achieve antioxidant effects has been widely reported in experimental studies. Liu et al. [82] extracted polysaccharides from *Lycium ruthenicum* by optimizing dynamic microwave-assisted extraction technology and tested their antioxidant activity in vitro. They found that polysaccharides were capable of scavenging DPPH, hydrogen peroxide, and superoxide anion radicals, and their activity of DPPH scavenging increased with increasing concentration. Skenderidis et al. found that the water extract of *L. barbarum* demonstrated free radical scavenging activity and inhibited DNA damage induced by peroxyl radicals in their study [83].

In addition, phenolic components are important secondary metabolites of natural products, and their antioxidant activities have been thoroughly studied [84]. In vitro, free radical scavenging and cell damage tests are commonly used to evaluate their antioxidant activity. Polyphenols have been found to alleviate oxidative stress-induced diseases such as cardiotoxicity and acute lung injury [85]. Catechins and other phenolic compounds can normalize lipid peroxidation and alleviate adriamycin-induced oxidative stress in rats [86]. Studies have shown that *lycium berry* polyphenols can increase the expression of antioxidant enzymes such as catalase, heme oxygenase-1, glutathione peroxidase, and SOD, as well as reduce the oxidative stress of lipopolysaccharide (LPS) stimulated acute lung injury mice [87].

### 4.2. LBPs Increase the Activity of Antioxidant Enzymes

Free radicals are atomic groups with unpaired electrons, which attack macromolecules in the human body and cause non-negligible damage to organisms [88]. ROS mainly include hydrogen peroxide (H_2_O_2_), superoxide anion (O2•−), singlet oxygen (1O_2_), hydroxyl radical (OH•), and hydroxyl ion (OH^−^) [88]. The body scavenges free radicals through oxidation-reduction by antioxidant enzymes, converting oxides into less toxic or harmless substances [89]. SOD, CAT, and GSH-Px can quickly eliminate oxygen free radicals and block the free radical chain reaction through interaction [89]. LBPs can regulate and enhance the activity of antioxidant enzymes to effectively remove free radicals [90]. Nuclear factor E2-related factor 2 (Nrf2) is a key regulator of antioxidant defense pathways [91]. LBPs can prevent oxidative stress by activating the Nrf2/ARE signaling pathway (see Figure 5) and activating the expression of genes encoding detoxification enzymes and antioxidant enzymes (such as heme oxygenase 1(HO-1), SOD2, CAT, and GSH) [92]. In the study of cyclophosphamide (CTX)-induced ovarian injury, LBPs upregulated the protein expressions of Nrf2, HO-1, and quinone oxidoreductase 1 and increased the activity of antioxidant enzymes in reducing oxidative stress damage [93]. In short, LBPs may improve the activity of antioxidant enzymes to protect tissues from oxidative damage.

### 4.3. LBPs Regulate Genes Related to Apoptosis, Ferroptosis, and Autophagy

Apoptosis is programmed cell death controlled by genes. The release of cytochrome c is a crucial step in apoptosis and is regulated by the B-cell lymphoma gene 2 (BCL2) family [94,95]. BCL2 is anti-apoptotic, while BAX is pro-apoptotic. The ratio of BCL2/BAX determines whether the cell initiates apoptosis [96]. LBPs can reduce apoptosis induced by oxidative stress by upregulating BCL2, downregulating BAX, and upregulating the activity of peroxidase to reduce the production of reactive oxygen species (see Figure 5). A study reported that [96] LBPs treatment reduced the apoptosis of and inhibited BAX in retinal pigment epithelium (RPE) cells and activated Bcl-2 in the hydrogen peroxidation-induced RPE oxidative damage model in turn protected RPE from oxidative stress damage. Alcohol causes excessive oxidative stress through processes such as increased malondialdehyde concentrations, generation of reactive oxygen species, and reduced antioxidant enzyme activity. LBPs could reverse it by regulating the balance between BAX and BCL2 [97]. In ischemia/reperfusion myocardial injury, LBPs therapy increased the rate of apoptosis and the concentration for SOD and P62 by activating the NRF2 signal to induce autophagy [98]. In addition, LBPs were associated with ferroptosis and autophagy. LBPs were considered as novel anticancer properties by triggering ferroptosis and might represent a therapeutic option for breast cancer. In this study, LBPs inhibited the activity and proliferation of human breast cancer cells, which were associated with high levels of ferroptosis [99]. Additionally, LBPs were protective against disease progression by regulating autophagy [98,100,101,102]. It was recently reported that LBPs played a direct role in suppressing pyroptosis. They inhibited the NLRP3 inflammasome in hyperoxia-induced acute lung injury and Aβ1-40 oligomer-induced RPE damage [103,104].

### 4.4. LBPs Can Regulate Mitochondrial Function

Mitochondria are continuously dynamic organelles that assume an indispensable role in cell homeostasis. Mitochondrial dysfunction leads to impaired cell survival and excessive production of ROS [105]. Excessive production of ROS exacerbates mitochondrial dysfunction and further deteriorates cell survival. PGC-1α, TFAM, and NRF-1 are three vital regulatory proteins in mitochondrial biogenesis [106,107]. LBPs can play antioxidant roles by regulating mitochondrial function. In the model of non-alcoholic fatty liver disease, LBPs treatment significantly reduced the intracellular lipid accumulation and concentrations of TG, alanine aminotransferase (ALT), aspartate transaminase (AST), and malondialdehyde, while increasing the concentrations of SOD, phospholipid hydroperoxides, GSH-Px, and CAT [108]. LBPs treatment up-regulated PGC-1 and further promoted the expression of TFRAM and NRF1, enhancing mitochondrial biogenesis and improving oxidative stress [108] (see Figure 5). Zhu et al. found that PM2.5 aggravated mitochondrial damage and induced cell apoptosis by upregulating the expression of pro-apoptotic BAX and downregulating the expression of anti-apoptotic BCL2, while LBPs could reverse this process to protect HaCaT cells [100]. In addition, LBPs inhibited the upregulation of GRK2, reduced the apoptosis of cardiomyocytes, and partially restored the imbalance of ischemia/reperfusion-induced mitochondrial division/fusion by regulating the expressions of Drp1, Opa1, and Mfn2 in AKT/eNOS signaling pathway in the ischemia/reperfusion injury model. A reduction in ROS production in cardiomyocytes was detected in vitro, which indicated the downregulation of cell apoptosis [109].

### 4.5. LBPs Alleviate Endoplasmic Reticulum Stress

The endoplasmic reticulum (ER) is the site of intracellular protein synthesis, folding, post-translational modification, and maintaining Ca^2+^ homeostasis [110]. The accumulation of misfolded proteins in organelles caused by external pathological factors is called endoplasmic reticulum stress (ERS) [111]. The occurrence of ERS is closely related to oxidative stress. ERS and oxidative stress can increase dysregulation of homeostasis, interfere with normal cell function, and activate pro-apoptotic signals through mutual positive feedback [112]. Glucose-regulated protein 78 (GRP78) and C/EBP homology protein (CHOP) expression were two notable markers of ERS [113]. Phosphorylated eukaryotic initiation factor-2 alpha (p-eIF2α) can reduce the transcription and translation of cells, thereby reducing protein production and regulating endoplasmic reticulum homeostasis [114]. LBPs have been shown in many studies to reduce oxidative damage by regulating endoplasmic reticulum stress (ERS). Yang et al. [115] found that LBPs intervention can improve ERS and oxidative stress, as well as increase the expression of antioxidase-related indexes in the testis of obese mice induced by the high-fat diet. Moreover, LBPs can ERS signaling by inhibiting the activation of p-eIF2α and GRP78-CHOP, reducing oxidative stress [115] (see Figure 5). In the oxidative stress injury of HaCaT cells induced by PM2.5 [100], the pretreatment of LBPs inhibited the expressions of intracellular GRP78 and CHOP, reduced the accumulation of apoptotic transcription factor CHOP in the nucleus, and regulated intracellular calcium ion homeostasis. These results suggested that LBPs reduced oxidative damage by inhibiting ER stress.

### 4.6. LBPs Can Promote Neuronal Regeneration in Oxidative Stress Damage

Some studies have shown that LBPs can counteract oxidative stress damage by promoting nerve regeneration. Oxidative stress shows an inhibitory effect in cavernosal nerve regeneration. A model of cavernosal nerve injury was developed by squeezing the cavernosal body, and continuous gavage of LBPs was conducted for 1 day, 7 days, and 14 days after surgery. The SOD and GSH-PX activities increased, and the serum MDA concentrations decreased in the treatment group. The number of myelinated axons in the cavernous nerve increased significantly in the treatment group 1 day after the injury, which reflected the nerve regeneration histologically. It suggested that LBPs effectively promoted nerve regeneration within two weeks before nerve injury to resist oxidative stress-induced neuronal damage [116]. In the study by Au et al., LBPs promoted intrinsic growth capacity and functional recovery of damaged neurons after severe peripheral nerve injury, as well as RGCs survival and axonal regeneration after optic nerve crush, which promoted the partial recovery of visual function [117].

## 5. Protective Role of LBPs in Oxidative Stress-Related Ocular Diseases

It is undeniable that LBPs have significant antioxidant effects. In ophthalmic diseases, the occurrence and development of many diseases are closely related to ROS. These diseases are called oxidative stress-related ocular diseases, including DR, hypertensive neuroretinopathy, AMD, RP, retinal ischemia/reperfusion injury, glaucoma, dry eye syndrome, and diabetic cataract. In the following, we will elaborate on the antioxidant effect of LBPs on improving oxidative stress-related ocular diseases (Table 1).

### 5.1. Diabetic Retinopathy

DR is one of the most severe microvascular complications in diabetic patients, and it is also a common cause of clinical blindness [146,147]. The pathological features of DR include the thickening of the retinal basement membrane, vessel occlusion, pathological neovascularization, ischemia of local tissue, and hypoxia, which cause visual impairment through oxidative stress injury, inflammatory injury, and other pathways [148,149]. Oxidative stress has been shown to stimulate the expression of pro-apoptotic molecules leading to apoptosis, which has been identified as one of the key mechanisms underlying cell damage in DR [150]. The antioxidant properties of LBPs facilitate their protective effects in animal models and cell lines of DR. However, no study has evaluated the effects of LBPs in patients with DR. In animal models of DR, LBPs administration ameliorated the structural and functional changes in the retina caused by diabetes [118,119,120]. Recovery of the thicknesses of the entire retina and each layer, reduction of structural disorders in the inner and outer segments of the photoreceptors, and decrease of cavitation in the RPE cell layer were observed after LBPs intervention. At the same time, a decrease in basement membrane thickness with the increase in the vessel thickness and morphologically normal capillaries was observed, suggesting a decrease in abnormal vascular clusters and tortuous capillaries with vascular proliferation. At a functional level, LBPs administration was associated with a decrease in the amplitude of a-waves, b-waves, and oscillatory potentials on electroretinography [121]. At the cellular level, the possible mechanism of LBPs in DR is possibly related to critical organelle: LBPs reduce oxidative stress in mitochondria and ER, which are two primary outcomes of diabetes-induced oxidative damage. Continuous exposure to ROS damages mitochondria and impairs the electron transport system, which ultimately leads to mitochondrial DNA damage and subsequent transcriptional impairment [151]. DNA damage and apoptosis of mitochondria are associated with changes in retinal blood flow and disruption of the blood-retinal barrier (BRB) during the late stages of DR [152]. Experimental studies have shown that LBPs may promote mitochondrial biogenesis by upregulating certain metabolic genes [122], which protects DR. Similarly, LBPs can attenuate ER stress, as hyperglycemia-associated oxidative stress disrupts protein synthesis and protein folding within the ER, ultimately leading to ER stress and subsequent apoptosis [118].

### 5.2. Hypertensive Neuroretinopathy

Hypertension increases systemic arterial pressure and peripheral vascular resistance and is a major risk factor for systemic vascular diseases. The fundus changes it causes include arteriolar stenosis, cotton patches, retinal hemorrhage, papilloma, and other microvascular and optic nerve abnormalities [153]. Microglia are the principal immunoreactive cells in the neurovascular system. Their overactivation induces harmful factors, including ROS and proinflammatory cytokines, and plays a vital role in the pathogenesis of hypertensive retinopathy [154]. LBPs were found to have protective effects on neurons and blood vessels in animal models of acute and chronic ocular hypertension. Some studies have found that LBPs can protect nerve function by moderately activating microglia or inhibiting the activation of the NLRP3 inflammasome, thereby regulating autophagy and MAPK pathways to improve microglia damage [123,124].

### 5.3. Age-Related Macular Degeneration

AMD is a leading cause of central visual impairment globally [155]. A recent population-based study showed that goji berries might help prevent or delay the development of AMD [125,126,127]. The main pathological changes associated with AMD are the damage of RPE cells, retinal warts and atrophy, and the obstruction of the choroidal capillary. The risk factors for these pathological changes include oxidative stress, inflammation, aging, and hypertension [156,157,158]. In in vitro studies, LBPs had anti-oxidative and anti-apoptotic effects on RPE cells [131]. In addition, other studies [96,130] on human RPE cell lines consistently concluded that the protective effect of LBPs on RPE cells was mainly due to their antioxidant effects, leading to the reduction of endogenous ROS concentrations. Similarly, a study reported that LBPs effectively protected photoreceptor cells from oxidative damage through antioxidant activities, thereby improving photoreceptor function in animal models [128]. LBPs intervention downregulated oxidative stress markers and upregulated the antioxidant genes Nrf2 and TrxR1 in an AMD animal model [129].

### 5.4. Retinitis Pigmentosa

RP refers to a group of different types of hereditary bilateral retinal pigment dystrophy characterized by the progressive loss of rod and cone photoreceptor cells, which eventually leads to total blindness [159]. Previous studies reported several mechanisms related to the pathogenesis of RP, including oxidative stress, ER stress, cyclic guanosine monophosphate signaling dysregulation, calcium accumulation, and inflammatory responses [160]. Among them, oxidative stress might be a crucial pathway for the retina to be highly susceptible to oxidative stress [161]. In a double-blind placebo-controlled trial involving 42 patients with RP [132], both visual acuity and mean macular thickness of RP patients after oral administration of LBPs were better than those of the control group, and the authors speculated that LBPs might help delay or reduce cone degeneration in RP. In studies involving a mouse model of RP [131,134], LBPs demonstrated a protective effect on the structure and function of retinal nerve cells through their antioxidant properties. The morphologies of photoreceptors and bipolar cells were restored, and the arrangement of photoreceptor cell layers was improved after LBPs supplementation. Functionally, photopic b-wave changes were detected by electroretinography, manifesting as shortened latency, increased amplitude, and increased scotopic a-and b-waves. Further investigation into the specific mechanism of photoreceptor protection revealed that increased antioxidant activity was observed in the animal model after LBPs administration, with a higher glutathione redox/antioxidant ratio, which was commonly used to determine the status of oxidative stress [133].

### 5.5. Retinal Ischemia/Reperfusion Injury

Retinal ischemia/reperfusion injury involves various pathological changes, including BRB destruction, glial cell activation, oxidative stress, and neuronal death [162]. There is limited research on LBPs and retinal ischemia/reperfusion injury. However, it was confirmed that LBPs improved retinal injury in the phenomenon/reperfusion injury animal model [135]. Animal models of middle cerebral artery occlusion were commonly used to study focal cerebral ischemia in the brain and evaluate the effect of LBPs in the retina with ischemia/reperfusion injury. In the present study, LBPs administered 1 week before ischemia protected the retina from nerve cell death, glial cell activation, oxidative stress, retinal swelling, and BRB disruption 48 h after reperfusion [135,137].

### 5.6. Glaucoma

Glaucoma is a group of ocular diseases that result in vision loss and blindness by damaging retinal ganglion cells [163]. Based on previous studies on the pathogenesis of glaucoma, increased oxidative stress is thought to undertake an essential role in the pathogenesis of glaucoma [164,165,166]. The concentration of free radicals is known to increase with cell aging, which impairs mitochondrial energy production and normal neuronal function [167]. In treating glaucoma, neuroprotective strategies, in addition to lowering intraocular pressure, are necessary to protect healthy neurons and rescue damaged neurons [168]. The neuroprotective effects [169] of LBPs and their ability to modulate oxidative stress [170] have been demonstrated to protect RGCs in both ocular hypertension-dependent [138,171] and -independent [139,140,172] optic neuropathy models. Chan et al. [142] first demonstrated the dose-dependent preservation of RGCs with LBPs pretreatment in an in vivo rat model of ocular hypertension induced by the protective effects of LBPs pretreatment on RGCs in injury models, including those of chronic ocular hypertension [123,138,143,171], acute ocular hypertension [136,139], partial optic nerve transection [140,173], and ischemia-reperfusion injury. Therefore, the benefits of LBPs pretreatment in experimental glaucoma models are evident.

### 5.7. Dry Eye Syndrome

DES is a disease of the eye surface that causes discomfort and visual impairment, affecting life quality [174]. DES is characterized by an abnormal tear film, eye discomfort, and potential damage to the ocular surface between the eyelids [175]. The treatment options for DES are limited to palliative care with artificial tears and tear preservation techniques. However, specific treatments may worsen endophthalmitis, despite the maximum use of palliative care, for the most severe cases of DES associated with corneal ulcers [176]. In a population meta-analysis of patients with DES, a Chinese herbal preparation containing wolfberry showed excellent potential for improving symptoms [177]. There are few studies on LBPs and dry eye disease. However, a study reported that LBPs administration played a noteworthy role in alleviating DES caused by oxidative stress and inflammation [144].

### 5.8. Diabetic Cataract

Diabetic cataract is also a complication secondary to diabetes, which is characterized by the gradual accumulation of glucose on the lens of the eye. Its pathogenesis is a complex process of multiple factors [21,57,178]. Sirtuin1 (SIRT1) is nicotinamide adenine dinucleotide (NAD) tower-dependent deacetylase that affects cell aging, differentiation, apoptosis, and the regulation of fat and glucose metabolism [179]. SIRT1 has been identified as a key regulator in cataract prevention. Previous studies showed that SIRT1 executed a role in regulating oxidative stress response and apoptosis in lens epithelial cells [180,181]. A recent cellular study in vitro explored the mechanism underlying the improvement of diabetic cataracts facilitated by LBPs. It showed that LBPs hindered the development of cataracts in the lens and improved retinal function by upregulating Sirt1 [145]. In previous studies, we found that SIRT1, which was highly sensitive to cellular redox states, counteracts the effects of ROS by deacetylation of multiple cellular targets. The redox function of cells was affected by SIRT1 directly or indirectly, and the activity and expression of SIRT1 were affected by the redox state of cells through post-translational modifications [182]. Thus, it is believed that SIRT1 is closely related to cellular redox.

## 6. Conclusions and Perspectives

In conclusion, LBP can directly or indirectly eliminate free radicals, including improving the activity of antioxidant enzymes, regulating apoptosis-related protein Bcl2/Bax, and enhancing mitochondrial function to eliminate free radicals, thus reducing the damage of oxidative stress on the body. Its mechanisms mainly involve Nrf2/ARE, Bcl2/Bax, PGC-1α/TFAM/NRF-1, and GRP78/CHOP/p-eif2α-related signaling pathway. Furthermore, its antioxidant effects show great potential in AMD, DR, hypertensive neuroretinopathy, RP, and other eye diseases. Moreover, as natural components, LBPs have shown potential for oxidative stress-related eye diseases. LBPs have the advantages of high production with low price and high safety. Notably, their efficacy would not be affected by the quality of wolfberry; they may be ideal candidates for the development of healthcare products and drugs.

However, the relationship between their structure and activity is complex, and their mechanisms of action in cells remain to be investigated. For instance, the mechanisms underlying their antioxidant role via the regulation of the enzyme-like SOD, CAT, and GSH-Px and non-enzyme-like lipoic acid, glutathione, L-arginine, and coenzyme Q10 remain to be further explored. Furthermore, it has not been established that LBPs can prevent oxidative stress by blocking epigenetic changes in DNA. The possible effects of LBPs in the post-genomic era are intriguing. The exploration of LBPs concerning genomics, transcriptomics, proteomics, and metabolomics may provide opportunities for drug engineering. In addition, the molecular structure and function of polysaccharides obtained by different extraction methods are quite different. Further exploration of the relationship between its structure and function, as well as its extraction process, will provide more insights into the applications of LBPs. With the development of analytical techniques, the separation and structural analysis of polysaccharides will provide insights into their underlying mechanisms. Further research also should focus on the specific protective mechanisms of LBPs in ocular cells. In addition, most LBPs are absorbed through the gastrointestinal tract by oral administration rather than directly acting on the eyes, which may influence the efficiency of LBPs on the eye. The extraction of active ingredients of *L. barbarum* and their direct action on the eyes, for example, by using modified nanomaterials, may present future research directions [183,184,185]. Currently, most studies on the effects of LBP on eye diseases are at the empirical stage, and some are in clinical trials. The results of the research are one-sided, leaving room for further exploration. In the future, the clinical application of traditional Chinese medicine compounds based on LBPs should be considered for specific ocular diseases with multicenter and large-sample clinical studies. Due to the prominent effects of LBPs on redox-related diseases, their dietary addition should be considered to improve the diet structure to facilitate disease treatment.

## Figures and Tables

**Table 1:** Reported studies evaluated LBP with oxidative stress-related ocular diseases.

SN	Authors (Year)	Study Subjects	Dosage of Administration	Results	Antioxidant Protection Pathway
Diabetic Retinopathy					
1	Tang et al. (2011) [118]	db/db mice.	Addition of 1% (kcal) *L. barbarum*, for 8 weeks.	LB ameliorated retinal abnormality in db/db type 2 diabetic mice.	LB attenuated endoplasmic reticulum stress and induced protein expression of AMPK, FOXO3α, and antioxidant enzymes thioredoxin and Mn SOD.
2	Hu et al. (2012) [119]	Rats by streptozotocin injection.	Administered LBPs (5 g/kg/d) orally by gavage for 8 weeks.	LBPs might have protective effects in diabetic retinopathy.	no report (NR)
3	Guo et al. (2013) [120]	Rats by streptozotocin injection.	LBP was given by gastrogavage for 24 weeks.	The ultrastructural changes were significantly alleviated by LBPs and confined to the inner nuclear layer.	LBPs could significantly alleviate pathological changes in the mitochondrion and inhibit neural cell apoptosis.
4	Yao et al. (2018) [121]	Rats by streptozotocin injection.	Pretreatment with LBPs in 2 concentrations (200, 400 mg/kg) for 20 weeks.	LBPs protected retinal function and morphology in diabetic rats.	LBPs might reduce neovascularization by re-establishing the balance between pro- and anti-angiogenic factors.
5	Yu et al. (2013) [122]	db/db mice.	Addition of 1% (kCal) LB, for 8 weeks.	Consumption of dietary LB could be beneficial to retinoprotection through the reversal of mitochondrial function in diabetic mice.	Mitochondrial pathway.
**Hypertensive neuroretinopathy**					
1	Chiu et al. (2009) [123]	Sprague–Dawley (SD) rats using argon laser photocoagulation.	Pretreatment with LBPs in 4 concentrations (1, 10, 100, 1000 mg/kg) for 4 weeks.	LBPs have been shown to be neuroprotective to RGCs against ocular hypertension (OH).	The neuroprotective effects of LBPs were partly due to modulating the activation of microglia.
2	Mi et al. (2020) [124]	Male SD rats were anesthetized with a mixture of ketamine and xylazine.	Administered LBPs (1 mg/kg) for 1 week.	LBPs could maintain the blood-retinal barrier and improve the survival rate of neurons in AOH injury.	LBPs regulated the production of amyloid-β and expression of receptors of advanced glycosylation end-products, as well as mediating the activity of retinal glial cells.
**Age-related macular degeneration**					
1	Bucheli et al. (2011) [125]	150 healthy subjects.	13.7 g/d of LB for 90 days.	LB protected from hypopigmentation and soft drusen accumulation in the macula of elderly subjects.	NR
2	Li et al. (2018) [126]	114 patients with early AMD.	25 g/day for 90 days.	LB improved macular pigment optical density in early AMD patients.	NR
3	Li et al. (2021) [127]	27 participants.	Consumed either 28 g of LB.	Regular intake of LB in a healthy middle-aged population increased macular pigment optical density and might help prevent or delay the development of AMD.	NR
4	Cheng et al. (2016) [128]	Male SD rats exposed to white light.	Diet supplemented with LBPs 250 mg/kg for 54 days.	LBPs had a protective effect on light-induced retinal degeneration via their antioxidant property.	LBPs could decrease MDA and increase total glutathione concentrations.
5	Tang et al. (2018) [129]	BALB/cJ mice exposed to white light.	Pretreatment with LBPs in 2 concentrations (150, 300 mg/kg) for 7 days.	Pretreatment with LBPs effectively protected photoreceptor cells against light-induced retinal damage probably.	LBPs eliminated oxygen radicals by upregulating the antioxidative genes Nrf2 and trxr1.
6	Liu et al. (2015) [96]	Human RPE cell line exposed to H_2_O_2_ for 24 h.	Pretreatment with LBPs in 6 concentrations (10, 50, 100, 500, 1000, 5000 ug/mL)	LBPs could protect ARPE-19 cells from H_2_O_2_-induced apoptosis.	The Bcl-2 family had a relationship with the protective effects of LBPs.
7	Hsieh et al. (2018) [130]	UVB irradiation-induced in ARPE-19 cells.	Pretreatment with LBPs in 2 concentrations (25, 50 ug/mL)	LBPs exhibited antioxidant activity and rescued UVB-induced apoptosis of ARPE-19 cells.	LBPs exerted a superior effect on rescuing, which might be associated with the activation of TLR signaling.
8	Liang et al. (2021) [131]	Human ARPE-19 cell line exposure to H_2_O_2_ for 24 h.	Treated with different concentrations of LBPs (0.25, 0.5, 1, and 2 mg/mL)	Pretreatment of ARPE-19 cells with LBPs exhibited high efficacy at reducing oxidative damage and inhibiting cell apoptosis.	LBPs might modulate the expression of proteins involved in the apoptotic pathway and activate the Nrf2 signaling pathway.
**Retinitis Pigmentosa**					
1	Chan et al. (2019) [132]	Forty-two RP subjects.	The daily dosage by oral 5 g net weight for 12 months.	LB supplement provided a neuroprotective effect for the retina and could help delay or minimize cone degeneration in RP.	NR
2	Wang et al. (2014) [133]	RD10 mouse, a photoreceptor fast-degenerating model of retinitis pigmentosa.	Administered LBPs (1 mg/kg) for 4 weeks.	LBPs protected rd10 mouse photoreceptors from a synergistic protective effect in degeneration.	LBPs modulated inflammation and apoptosis partly through inhibition of NF-κB and HIF-1α expressions, respectively.
3	Liu et al. (2018) [134]	RD1 mouse, a photoreceptor fast-degenerating model of retinitis pigmentosa.	Administered LBPs (10 mg/kg).	LBPs improved retinal morphology and function in rd1 mice and delayed the functional decay of RGCs during photoreceptor degeneration.	NR
**Retinal Ischemia/Reperfusion Injury**					
1	Li et al. (2011) [135]	Inserting fibers coated with vinyl polysiloxane into the right internal carotid artery of mice.	Administered LBPs (1 mg/kg) for 1 week.	LBPs effectively alleviated ischemia-induced retinal dysfunction as well as reduced correlated neuronal death and glial activation.	LBPs increased the number of calretinin-positive cells, enhanced protein kinase Cα immunoreactivity, and attenuated glial fibrillary acidic protein expression.
2	He et al. (2014) [136]	Male SD rats were anesthetized with a mixture of ketamine and xylazine.	Administered LBPs (1 mg/kg) for 2 weeks.	LBPs had a protective effect on retinal ischemia-reperfusion(I/R) injury.	LBP partially exerted its beneficial neuroprotective effects via the activation of Nrf2 and increased HO-1 protein expression.
3	Yang et al. (2017) [137]	Inserting fibers coated with vinyl polysiloxane into the right internal carotid artery of mice.	Pretreatment with LBPs in 2 concentrations (1, 10 mg/kg) for 7 days.	LBPs might have a neuroprotective role to fulfill in ocular diseases for which I/R was a feature.	LBPs protected the retina from neuronal death, apoptosis, glial cell activation, aquaporin water channel up-regulation, disruption of BRB, and oxidative stress.
**Glaucoma**					
1	Mi et al. (2012) [138]	SD rats using argon laser photocoagulation.	Pretreatment with LBPs in 1 concentration (1 mg/kg) for 3 weeks.	The neuroprotective effect of LBPs on RGCs might be related to their ability to regulate the endothelin-1(ET-1)-mediated biological effects on RGCs and retinal vasculature.	Neuroprotective effects of LBPs might be related to regulating the endothelin system.
2	Mi et al. (2012) [139]	Mouse model induced in unilateral eye by introducing 90 mmHg ocular pressure.	Pretreatment with LBPs in 1 concentration (1 mg/kg) for 7 days.	LBPs could prevent damage to RGCs from Acute ocular hypertension (AOH)-induced ischemic injury.	Effects on blood vessel protection of LBPs.
3	Chu et al. (2013) [140]	SD rat model with partial optic nerve transection (PONT).	Administered LBPs (1 mg/kg) via a nasogastric tube for 4 weeks.	LBPs altered the functional reduction caused by PONT by regulating the signal from the outer retina.	NR
4	Li et al. (2013) [141]	SD rat model with (PONT).	Administered LBPs (1 mg/kg) for 4 weeks.	LBPs could delay secondary degeneration in the CNS by modulating the function of microglia/macrophages.	LBPs could delay secondary degeneration of the axons and inhibit the activation of microglia/macrophages.
5	Chan et al. (2007) [142]	Ocular hypertension model in rats by laser photocoagulation.	Administered LBPs (1 mg/kg).	LBPs could improve retinal nerve degeneration in a rat model of ocular hypertension.	NR
6	Lakshmanan et al. (2019) [143]	Rat model of chronic ocular hypertension.	Administered LBPs (1 mg/kg) for 11 weeks.	LBPs posttreatment arrested the subsequent neuronal degeneration after treatment commencement and preserved RGCs density and retinal functions in a chronic OHT model.	NR
**Dry Eye Symptom**					
1	Chien et al. (2018) [144]	Male SD rats.	Pretreatment with LBPs in 3 concentrations (250, 350, 500 mg/kg) for 21 days.	LBPs might have important effects in alleviating dry eye disease induced by oxidative stress and inflammation.	NR
**Diabetic cataract**					
1	Yao et al. (2020) [145]	Human lens epithelial cell line SRA01/04 cells were cultured under the high glucose medium.	Pretreatment with LBPs in 3 concentrations (100 mg/L, 200 mg/L, 400 mg/L)	LBPs prevented diabetic cataracts in animals by upregulating Sirt1 and Bcl-2 and suppressing cell death-related genes.	LBPs regulated genes related to cell death and reduced oxidative stress.
2	Yao et al. (2020) [145]	Diabetes mellitus was induced in rats by streptozotocin injection	Pretreatment with LBPs in 2 concentrations (25 mg/kg, 50 mg/kg), for 8 weeks.	LBPs alleviated the progression of diabetic cataracts in the rat.	LBPs regulated genes related to cell death and reduced oxidative stress.

## Data Availability

Data sharing not applicable.
